# Impaired Hypothalamic mTOR Activation in the Adult Rat Offspring Born to Mothers Fed a Low-Protein Diet

**DOI:** 10.1371/journal.pone.0074990

**Published:** 2013-09-09

**Authors:** Omar Guzmán-Quevedo, Raquel Da Silva Aragão, Georgina Pérez García, Rhowena J. B. Matos, André de Sa Braga Oliveira, Raul Manhães de Castro, Francisco Bolaños-Jiménez

**Affiliations:** 1 Unité Mixte de recherche 1280 Physiologie des Adaptations Nutritionnelles, Institut National de la Recherche Agronomique, Nantes, France; 2 Université de Nantes, Nantes Atlantique Université, Nantes, France; 3 Núcleo de Educação Física e Ciências do Esporte, Universidade Federal de Pernambuco, Vitória de Santo Antão, Pernambuco, Brazil; 4 Departamento de Nutrição, Centro de Ciências da Saúde, Universidade Federal de Pernambuco, Recife, Pernambuco, Brazil; University of Arkansas for Medical Sciences, United States of America

## Abstract

Several epidemiological and experimental studies have clearly established that maternal malnutrition induces a high risk of developing obesity and related metabolic diseases in the offspring. To determine if altered nutrient sensing might underlie this enhanced disease susceptibility, here we examined the effects of perinatal protein restriction on the activation of the nutrient sensor mTOR in response to acute variations in the nutritional status of the organism. Female Wistar rats were fed isocaloric diets containing either 17% protein (control) or 8% protein (PR) throughout pregnancy and lactation. At weaning offspring received standard chow and at 4 months of age the effects of fasting or fasting plus re-feeding on the phosphorylation levels of mTOR and its downstream target S6 ribosomal protein (rpS6) in the hypothalamus were assessed by immuno-fluorescence and western blot. Under *ad libitum* feeding conditions, PR rats exhibited decreased mTOR and rpS6 phosphorylation in the arcuate (ARC) and ventromedial (VMH) hypothalamic nuclei. Moreover, the phosphorylation of mTOR and rpS6 in these hypothalamic nuclei decreased with fasting in control but not in PR animals. Conversely, PR animals exhibited enhanced number of pmTOR imunostained cells in the paraventricular nucleus (PVN) and fasting decreased the activation of mTOR in the PVN of malnourished but not of control rats. These alterations occurred at a developmental stage at which perinatally-undernourished animals do not show yet obesity or glucose intolerance. Collectively, our observations suggest that altered hypothalamic nutrient sensing in response to an inadequate foetal and neonatal energetic environment is one of the basic mechanisms of the developmental programming of metabolic disorders and might play a causing role in the development of the metabolic syndrome induced by malnutrition during early life.

## Introduction

Many epidemiological and experimental studies have demonstrated that a deficient or excessive provision of nutrients during *in utero* development and/or neonatal life increases the risk of developing the metabolic syndrome in adulthood. Actually, the rate of obesity, hypertension and insulin resistance is higher among individuals born at low birth weight than those of normal weight [1] and the offspring of dams exposed to nutrient restriction or overfeeding during pregnancy and/or lactation exhibit several physiological disturbances linked to the metabolic syndrome including insulin resistance [2]–[4], reduced leptin sensitivity [5], hepatic steatosis [6], hypertension [7], [8] and hyperlipidemia [2], [9]. These observations have been explained by the thrifty phenotype and the Developmental Origins of Health and Disease (DOHaD) hypothesis [10], [11].

Several tissue and cellular dysfunctions have been proposed as underlying mechanisms of the developmental programming of the metabolic syndrome including altered organ structure [12], [13], elevated glucocorticoids and endocrine sensitivity [14], [15], impaired mitochondrial function [16], [17], altered feeding behaviour [18], [19] and impaired gene expression resulting from the detrimental effects of perinatal malnutrition on the epigenome [20]–[22]. Strikingly, maternal nutrient restriction, maternal obesity, neonatal overfeeding or neonatal malnutrition, all result in common physio-pathological outcomes in adulthood [21], [23], [24]. This has led to suggest that sub-optimal or excessive calorie intake during early development affect a common set of genes that might act as gatekeepers of a fundamental gene network or signalling pathway such that disturbances in the expression of only a small number of these gatekeeper genes may have a major impact on cell metabolism and energy homeostasis [25]. At the present time these putative gatekeepers are unknown, but it has been proposed that they might be transcription factors as well genes involved in the regulation of the epigenetic machinery or cell/tissue differentiation [25].

In a previous genome-wide study aimed to get an integrated view of the molecular pathways that might underlie the feeding perturbations associated with metabolic programming, we observed that perinatal under-nutrition induces permanent changes in the transcriptional profile of several genes which act as gatekeepers for regulation of nutrient sensing [26]. Notably, the expression of phosphatidylinositol 3-kinase (PI3K) and the serine threonine kinase AKT were significantly increased in the hypothalamus of adult rats born to protein-restricted dams [26]. AKT is an upstream regulator of the mammalian target of rapamycin (mTOR). This latter kinase acts as nutrient sensor of high nutrient supply in different peripheral tissues where it regulates positively protein synthesis and lipogenesis [27]. mTOR is also involved in the control of feeding behaviour by integrating hormonal and nutrient signals in the hypothalamus [28] and, therefore, regulates energy homeostasis at the whole body level.

Based on these observations, we hypothesized that central adaptation of nutrient sensing to an inadequate foetal and neonatal energetic environment might be one of the basic mechanisms of the developmental programming of metabolic disorders. To test this hypothesis, here we analysed the impact of perinatal protein restriction on the activation of mTOR and its downstream target S6 ribosomal protein (rpS6) in the hypothalamus in response to fast and to fasting plus re-feeding.

## Materials and Methods

### Ethics Statement

All experiments were performed in accordance with the European Communities Council Directive of 24 November 1986 (86/609/EEC) and the Principles of laboratory animal care (NIH publication no. 85–23, revised 1985), and were approved by the Ethical Committees for Animal Experimentation of the Pays de la Loire (protocol CEEA.2012.15), and of the Federal University of Pernambuco (CEUA, protocol 23076.026942/2010-10). All efforts were taken to prevent and ameliorate any suffering during the experiments and animals were sacrificed using a rising concentration of CO_2_ and cervical dislocation.

### Animals and nutritional manipulations

Virgin female Wistar rats weighing 200–220 g were obtained from Charles River (France) and placed under a 12 h light/dark cycle (lights on at 7 A.M.), with food and water *ad libitum* for at least eight days before any experimental manipulation. They were then paired with males of the same strain and age. After confirmation of mating, pregnant rats were housed individually and fed either a control (170 g protein/kg) or an isocaloric low-protein diet (80 g protein/kg) until the end of lactation. Both diets were prepared in the Department of Nutrition of the Federal University of Pernambuco according to the recommendations of The American Institute of Nutrition for growth and reproduction of rodents [29] and their composition has been described previously [30]. One day after delivery, litter size was adjusted to eight pups per dam with an equal sex ratio. At weaning (21 days), only male rats remained in the study and all control (C) and protein-restricted (PR) pups were onto standard chow. At 4 months of age, animals were sacrificed under *ad libitum* feeding conditions or after a fasting period of 48 h. A third set of rats from each control and protein-restricted groups was food-deprived for 48 h and re-fed for 3 h before sacrifice. The *ad libitum* fed group was fasted for one hour before sacrifice to attain a homogenous basal level of mTOR activity across the different animals and all rats were sacrificed within the first hour after the beginning of the light cycle.

### Hormone and metabolite determinations

Insulin resistance was evaluated 15 days before sacrifice by the glycemic response to an oral glucose tolerance test. After a fast of 16 h, animals were given an oral dose of 1 g/kg of glucose and a drop of blood was taken immediately before and 20, 40, 60, 80, and 120 min after the administration of glucose by severing the tip of the tail. Blood glucose was determined with a blood glucose monitor (Accu-Check® Active, Roche Diagnostics). Plasma from trunk blood collected at the time of sacrifice was assayed for insulin and leptin using assay kits from Millipore (Billerica, MA). Enzymatic methods were used to quantify the levels of triglycerides (kit PAP 150, BioMérieux, Craponne, France), glucose (kit PAP 1200, BioMérieux), and cholesterol (Cholesterol RTU, Biomerieux). Free fatty acids were evaluated using an assay kit from Wako (Richmond, VA).

### Western blot analysis

Following sacrifice, the hypothalamus was excised, immediately frozen in liquid nitrogen and stored at −80°C until processing. For Western Blot analysis, 100 µg of protein extracts were separated with 10% SDS-PAGE gel and transferred onto PVDF membranes. Nonspecific binding was blocked by incubating the membranes for 1 h at room temperature with 5% BSA dissolved in TBST (pH 7.5, 10 mM Tris–HCl, 150 mM NaCl, and 0.1% Tween-20). The membranes were then incubated overnight at 4°C with primary antibodies detecting the endogenous levels of AKT, mTOR, rpS6 or their phosphorylated forms at, respectively, ser 473, ser 2448 and ser 235/236. All antibodies were used at a dilution of 1∶1000 in blocking buffer and were purchased from Cell Signaling (Danvers, MA, USA). Afterwards, membranes were incubated for 1 h at room temperature with peroxidase-conjugated secondary antibodies (Jackson ImmunoResearch, West Grove, PA). To control for protein loading, the same blots were incubated with a β-Actin antibody (Sigma-Aldrich, St Louis, MO, USA), diluted at 1∶500. Immunoblotted proteins were visualized with the UptiLight™ HRP blot substrate (Interchim, France) and analyzed using the G:BOX Chemi XL system (Syngene, Cambridge, UK).

### Immunofluorescence

After deep anaesthesia with sodium pentobarbital, animals were transcardially perfused with phosphate-buffered saline (PBS) followed by 4% paraformaldehyde in PBS. The brains were dissected from the skulls, post-fixed for an additional period of 4 hours in the same fixative and cryoprotected by immersion in 30% sucrose/PBS for 48 hours. Coronal sections (50 µm), through the entire extend of the hypothalamus were obtained with the use of a cryostat.

Free floating sections were incubated at room temperature in blocking buffer (5% Normal Donkey Serum, 0,3% Triton X-100, in PBS), for 1 h followed by overnight incubation at 4°C with rabbit polyclonal antibodies raised against the phosphorylated forms of mTOR or rpS6 at a dilution of 1∶50. The next day, sections were washed 4×5 min with PBS and incubated for 2 h in the dark at RT with biotinylated secondary antibodies. Sections were then incubated for 1 h in the dark with strepavidin-conjugated Alexa 568, washed, mounted onto slides and covered under Vectashield (Vector Laboratories, Burlingame, CA). For double labelling, sections were processed for pmTOR labelling as described before followed by the incubation at 4°C overnight with a rabbit anti- Pro-opiomelanocortin (POMC) antibody (Phoenix Pharmaceuticals, Burlingame, CA) at a dilution of 1∶2000. After washing, sections were incubated for 1 h in the dark with goat anti-mouse Alexa 488, rinsed, mounted onto slides and covered under Vectashield.

### Image analysis and quantification

All the slides were analyzed by an investigator blind to the nutritional status of the animals. Single and double labelled sections were analyzed and documented using a fluorescence microscope attached to an Axioplan imaging Apotome apparatus (Carl Zeiss S.A.S., Le Pecq, France). Processing of digital images was performed using the softwares AXIOVISION (Carl Zeiss) and the number of immunopositive cells was automatically determined using automated image analysis software (Volocity®). At least three slices per animal spanning the mediobasal hypothalamus, including the arcuate nucleus (ARC) and the ventromedial hypothalamic nucleus (VMH), or the paraventricular nucleus (PVN) were used to quantify the number of labelled cells. To determine the number of POMC-positive cells expressing pmTOR, six to eight areas per slice were analyzed based on the number of immunostained nuclei and their uniform fluorescence intensity. 8–12 optical sections (1 µm thick) were scanned from each area using the 32× objective. Cells were scored as double-labelled when the POMC labelling was unambiguously associated with a pmTOR-positive cell in stack of sections. As for quantification of single-labeled cells, images of double-labeled tissue sections were digitized, and areas of interest were identified and outlined according to the stereotaxic atlas of Paxinos and Watson.The total number of immunopositive cells in all the sections from a given animal was divided by the number of examined sections to determine the mean number of immunostained cells per rat.

### Statistical analysis

Statistical differences between control and PR rats under *ad libitum* feeding conditions were assessed by unpaired Student's t-test. To assess the effects of protein restriction on the activation of mTOR in response to fasting and to fasting plus re-feeding in each group, data were analyzed by one-way ANOVA followed by Dunnett's Multiple Comparison Test. Statistical significance was set at P<0.05.

## Results

### Phenotypic characteristics of the pups and metabolic variations in response to fasting

**Figure 1:**
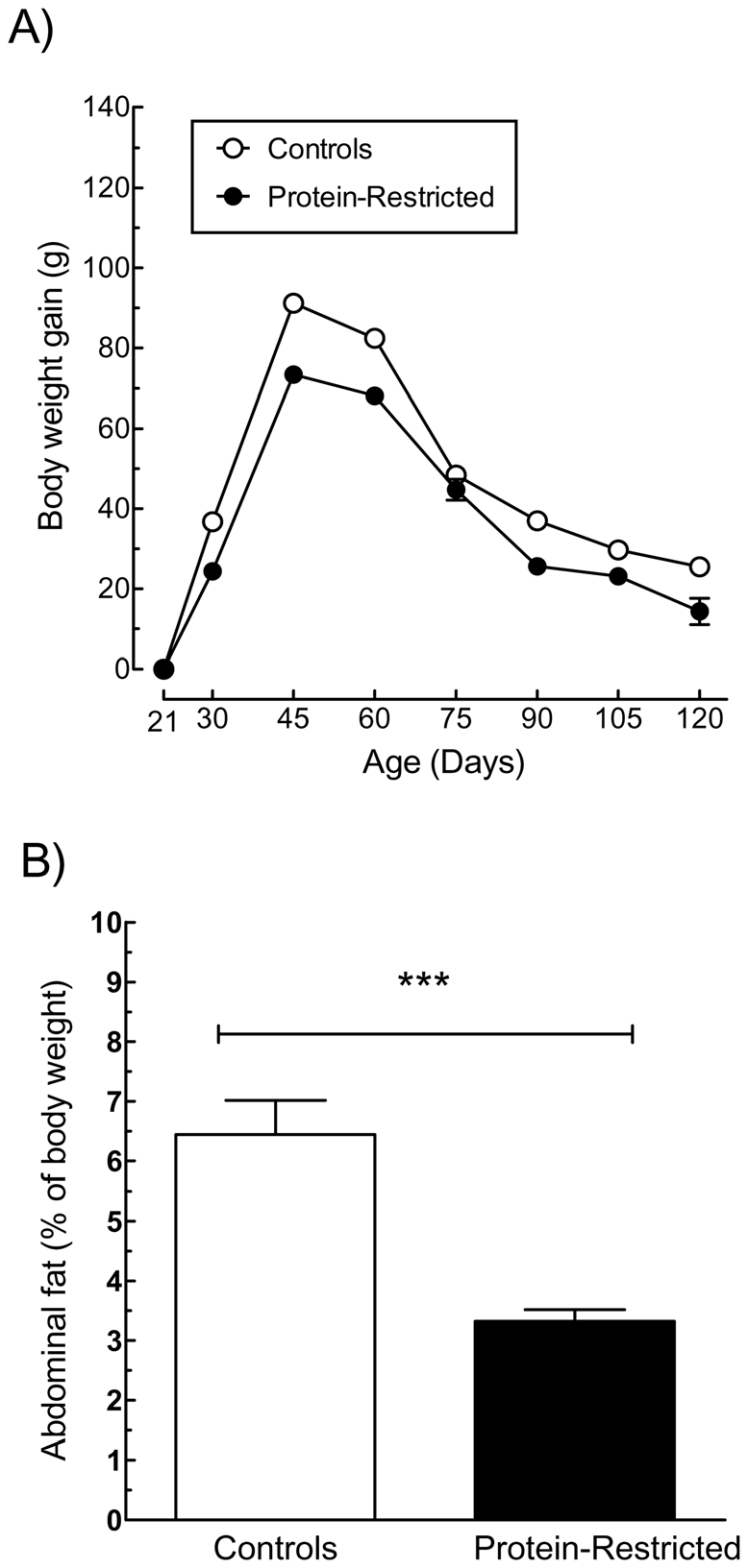
Effects of early protein-restriction on body weight characteristics. Graphs illustrate post-weaning growth pattern (A) and body fat mass at adulthood (B) of offspring born to control or protein-restricted dams. Growth pattern is expressed as the amount of body weight gain in grams between the indicated periods of time. Abdominal fat refers to the summed weight of bilateral fat depots from 3 regions (inguinal, retroperitoneal and epididymal) and the mesenteric fat pad at 4 months of age expressed in percentage of body weight. ***P<0.001 (Student's t-test with n = 18 animals per group).

**Figure 2:**
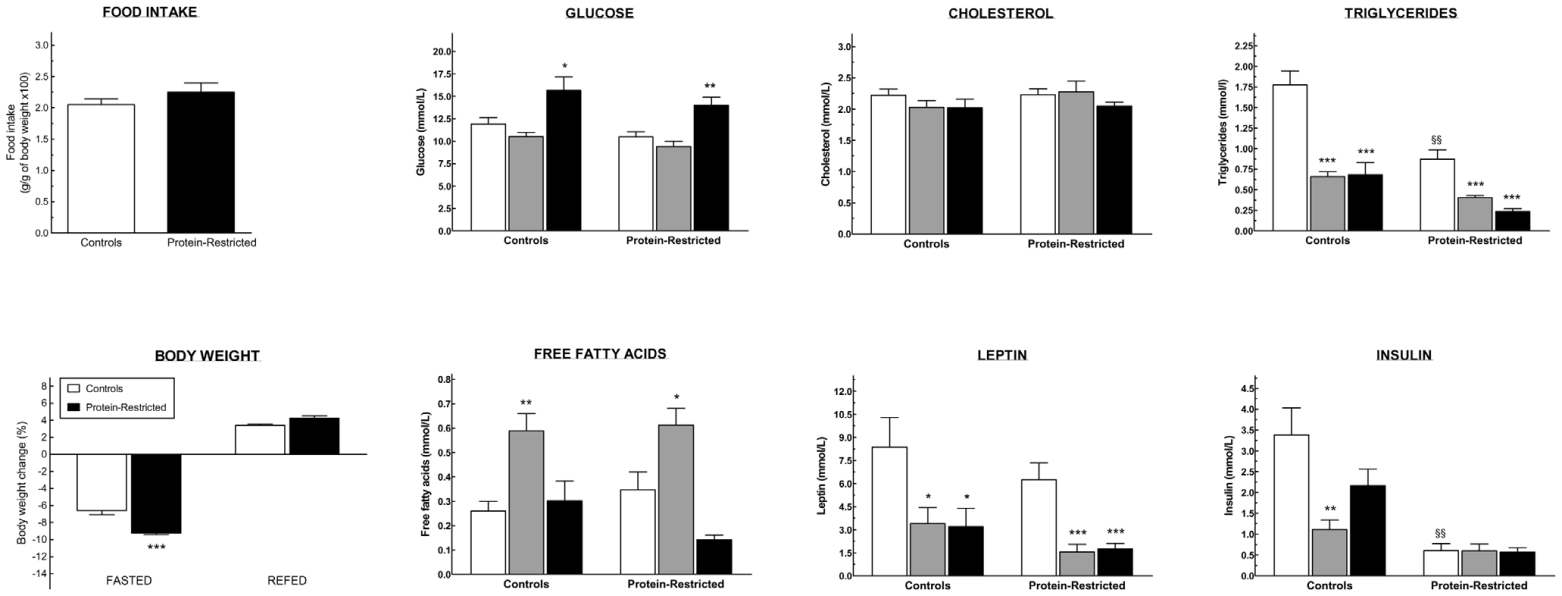
Changes in food intake, body weight and serum hormone and metabolite levels in response to variations in nutrient supply in adult rats born to control or protein-restricted dams. Animals were sacrificed under *ad libitum* feeding conditions or food-deprived with free access to water for a period of 48 h after which they were either sacrificed or fed for 3 h and sacrificed immediately after. *P<0.05; **P<0.01; ***P<0.001 in comparison to *ad libitum* fed animals of the same experimental group as determined by One way ANOVA followed by Dunnett's test. ^§§^p<0.01 in comparison to *ad libitum* fed control animals as determined by Student's t-test. A minimum of 5 animals per group was used for the determinations.

In agreement with our previous observations [31], the offspring born to protein-restricted dams exhibited the same pattern of growth than control animals (Figure 1A), along with lower body weight (C = 340±5.02 g vs LP = 255±5.42 g, ***p<0.0001), reduced abdominal fat (Figure 1B), and decreased plasmatic levels of triglycerides and insulin in the fed state (Figure 2). Otherwise, we observed no differences in the plasmatic content of glucose, cholesterol, free fatty acids or leptin between both groups under *ad libitum* feeding conditions. Moreover, control and PR rats displayed the same glucose tolerance as shown by the identical area under the curve of the glucose tolerance test and the same temporal decrease in plasmatic glucose after the glucose load (Figure S1).

After fasting for 48 h, the plasmatic levels of triglycerides, leptin and insulin decreased significantly in control rats and these changes were associated with a concomitant increase in the concentration of free fatty acids that returned to pre-fasting levels after re-feeding (Figure 2). Fasted PR rats exhibited also reduced plasmatic levels of triglycerides and leptin and increased free fatty acids in relation to PR rats fed *ad libitum* (Figure 2). However, fasting did not alter the plasmatic content of insulin in PR rats (Figure 2), and after re-feeding the levels of free fatty acids tended to decrease below those exhibited by *ad libitum* fed PR animals. These latter differences cannot be attributed to variations in food intake between control and PR rats because both groups consumed the same amount of food during the re-feeding period (Figure 2). Moreover, the reduction in body weight after fasting was more pronounced in PR rats (Figure 2) suggesting that the compensatory changes of energy expenditure during fasting are impaired in PR animals. In addition, the fact that the offspring born to protein-restricted dams exhibited the same pattern of growth than control animals (Figure 1A), indicates that the differences in body weight and other phenotypic parameters exhibited by PR rats in response to fasting, are not the result of a compensatory mechanism to overcome their initial state of udernutrition but the direct consequence of their exposure to a low-protein diet during gestation and lactation.

### Effect of perinatal protein restriction on the hypothalamic activation of mTOR

**Figure 3:**
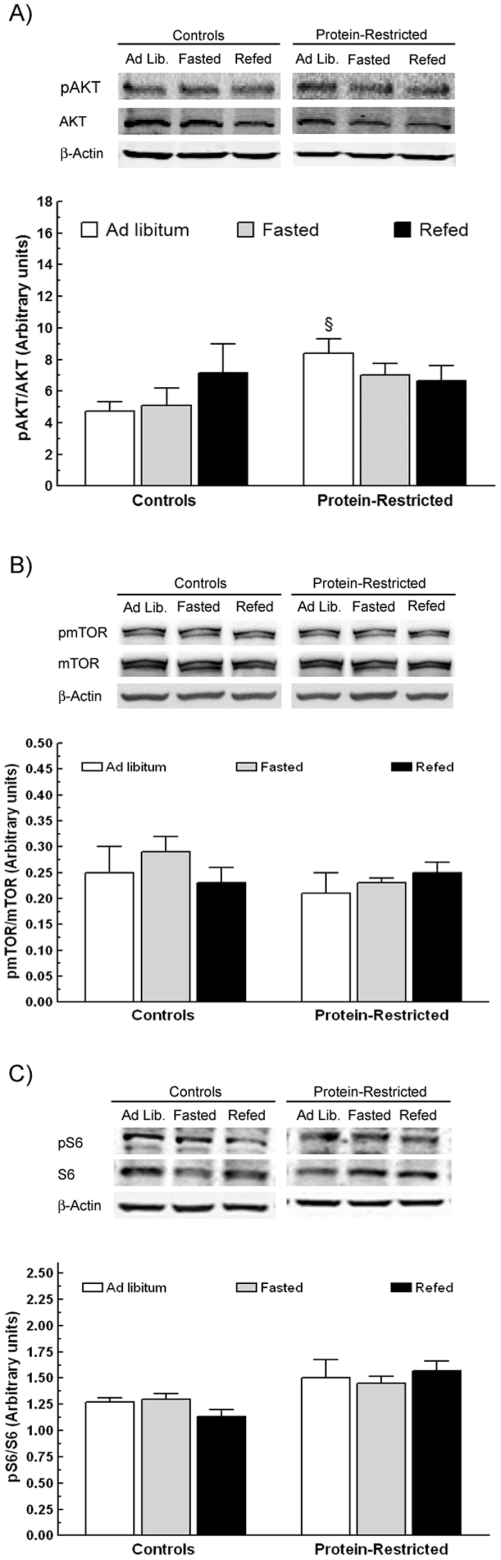
Effect of early protein restriction on the mTOR signaling pathway. Graphs illustrate the activation of AKT (A), mTOR (B) and S6 ribosomal protein (C) in the hypothalamus in response to variations in nutrient supply in adult rats born to control or protein-restricted dams as determined by western blot analysis. The figures above each histogram correspond to representative western blots of the phosphorylated and non phosphorylated forms of the indicated proteins as well as of β-actin which was used as internal control of protein loading. Animals were sacrificed under ad libitum feeding conditions, after a fasting period of 48 h or after fasting followed by re-feeding for 3 h. ^§^P<0.05 compared to *ad libitum* fed controls animals.

On the base of our previous observations showing increased expression of hypothalamic AKT in protein-restricted rats [26], we first performed a series of western blot experiments to determine the phosphorylation levels of AKT. The results of these experiments indicated enhanced activity of AKT in protein-restricted animals under *ad libitum* fed conditions (Figure 3A). However, we observed no change in the phosphorylation levels of AKT in response to fasting or to fasting plus re-feeding neither in control nor in PR animals (Figure 3A).

To test whether the nutrient sensor mTOR could be submitted to metabolic programming, we then examined by western blot the phosphorylated levels of mTOR and S6 ribosomal protein in control and PR animals exposed to acute food deprivation or to fasting plus re-feeding. Using this experimental approach, we did not find significant differences between the two groups in the activity of mTOR as indicated by the identical ratios of the phosphorylated versus the non phosphorylated forms of mTOR and rpS6 (Figure 3B and 3C).

**Figure 4:**
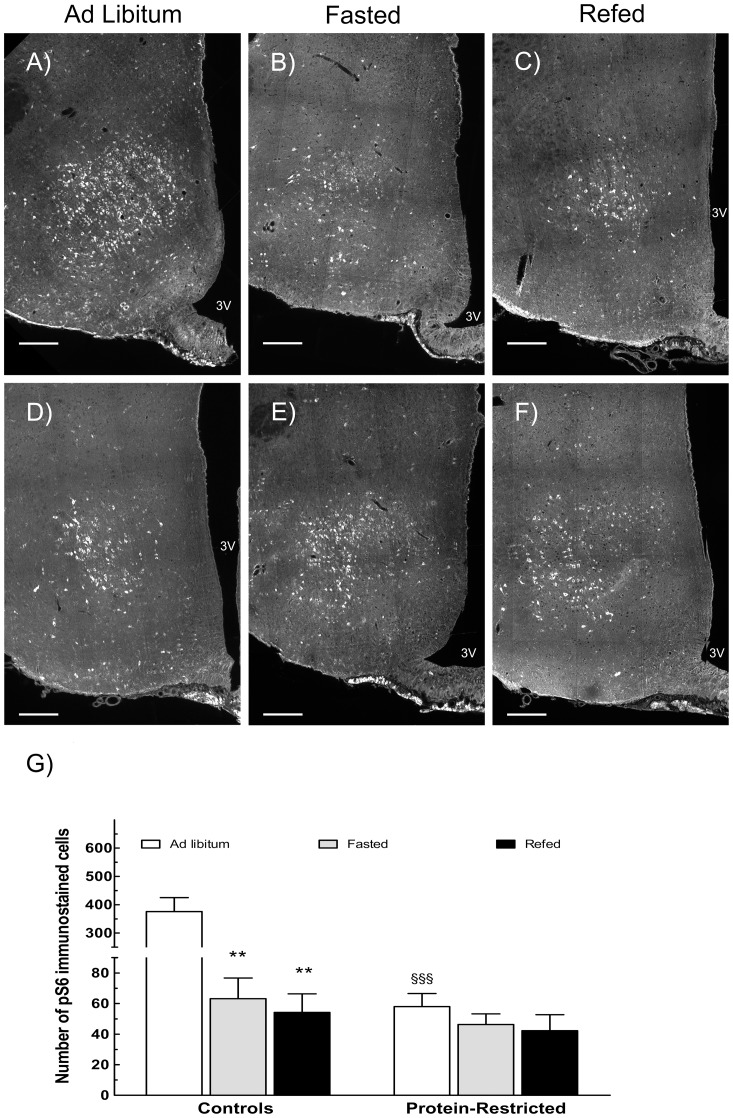
Changes in the phosphorylation status of S6 ribosomal protein within the mediobasal hypothalamic nucleus in response to variations in nutrient supply in adult rats born to control or protein-restricted dams. Top panels correspond to representative images of phosphorylated rpS6 labeling in hypothalamic slices from control (A,B,C) or perinatally undernourished rats (D,E,F) sacrificed under *ad libitum* feeding conditions, after a fast of 48 h or after fasting followed by re-feeding. Bottom graph (G), corresponds to the mean number of phosphorylated rpS6 immune-positive cells in the fed state or after fasting and fasting plus re-feeding. ^§§§^p<0.001 as compared to *ad libitum* fed control animals (Student's t-test). **P<0.01 in relation to *ad libitum* fed animals from the same experimental group (One way ANOVA followed by Dunnett's test). n = 5 for all groups. Scale bar  = 200 µm for all pictures. 3 V, third ventricle.

We then evaluated the number of hypothalamic cells immuno-stained with antibodies recognizing the total or the phosphorylated forms of mTOR and rpS6. In line with the results of Western blot, this analysis revealed no differences between control and PR rats in the number of mTOR or rpS6 expressing cells in the arcuate and ventromedial hypothalamic nuclei nor in the number of mTOR positive cells in the PVN. However, PR rats exhibited a significant reduction in the number of phosphorylated rpS6-labelled cells in the VMH under *ad libitum* feeding conditions in relation to their control counterparts (Figure 4A, 4D and 4G). Moreover, whereas control animals exhibited a significant reduction in the number of phosphorylated S6 ribosomal protein immunostained cells with food deprivation and re-feeding (Figure 4A, 4B, 4C and 4G), perinatally undernourished animals exhibited the same level of rpS6 phosphorylation in response to these nutritional manipulations (Figure 4D,4E,4F and 4G). We observed identical variations in the pattern of pmTOR staining to fasting and to fasting plus refeeding both in control and PR rats (Figure S2), indicating that, under our experimental conditions, the level of mTOR phosphorylation can be used as a reliable readout of mTOR activity.

**Figure 5:**
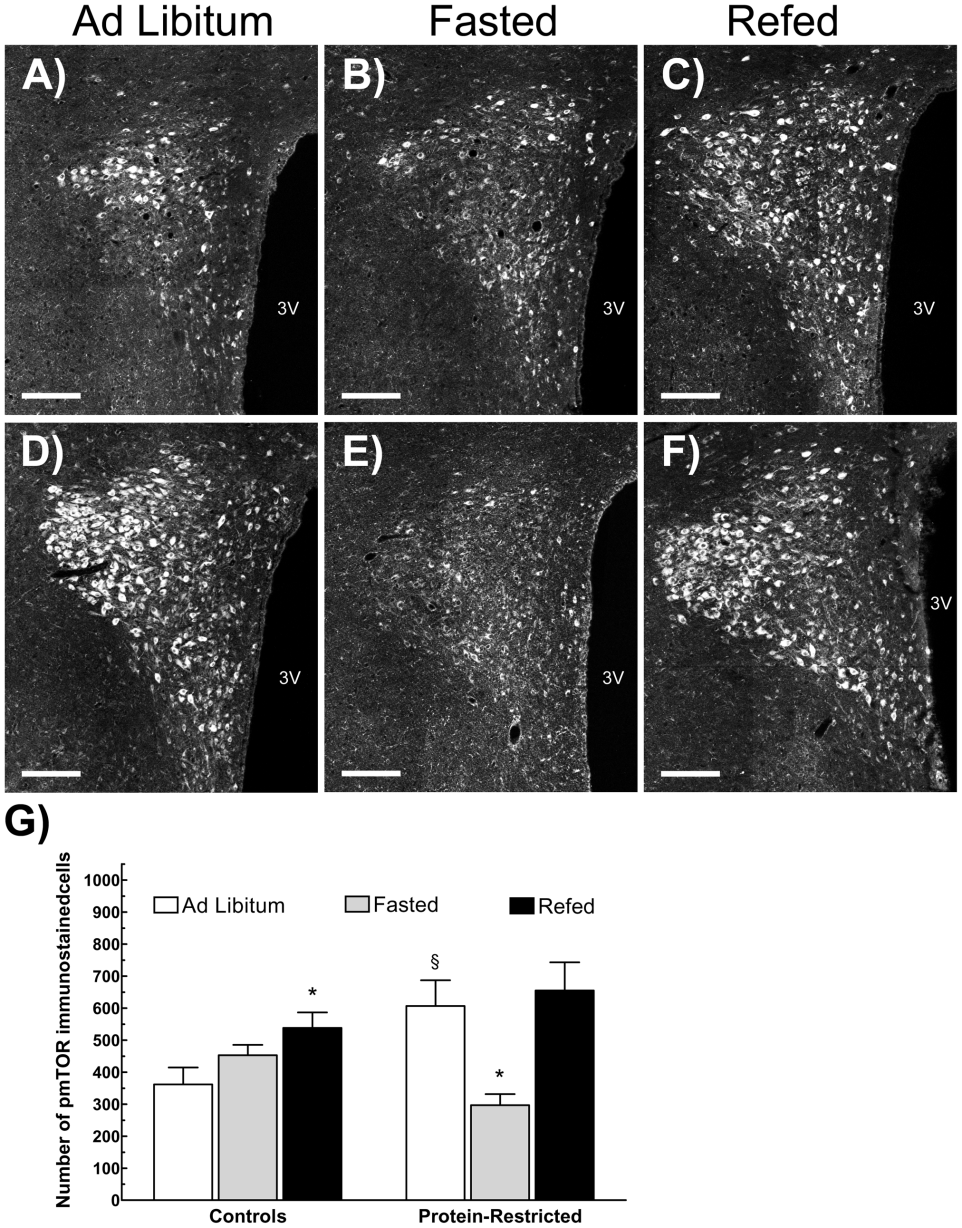
Changes in mTOR phosphorylation within the paraventricular nucleus of the hypothalamus in response to variations in nutrient supply in adult rats born to control or protein-restricted dams. Top panels correspond to representative images of pmTOR labeling in hypothalamic slices from control (A,B,C) or perinatally undernourished rats (D,E,F) sacrificed under *ad libitum* feeding conditions, after a fast of 48 h or after fasting followed by 3 h of re-feeding. Bottom graph (G) corresponds to the mean number of immune-positive pmTOR cells in the fed state or after fasting and fasting plus re-feeding. 3 V, third ventricle. ^§^p<0.05 as compared to *ad libitum* fed control animals (Student's t-test). *P<0.05 in relation to *ad libitum* fed animals from the same group (One way ANOVA followed by Dunnett's test). n = 5 for all groups. Scale bar  = 300 µm for all pictures.

In contrast to the observations in the ARC and VMH, under *ad libitum* feeding conditions PR rats exhibited a significant increase in the number of cells expressing pmTOR in the PVN in relation to controls (Figure 5A, 5D and 5G). In addition, while fasting induced no change in the number of pmTOR-immunopositive cells in control rats (Figure 5A, 5B and 5G), the same stimuli decreased the phosphorylation levels of mTOR in PR animals (Figure 5D, 5E and 5G). Moreover, control rats exhibited enhanced activation of mTOR after re-feeding that overstepped the one observed under *ad libitum* feeding conditions (Figure 5C and 5G). In contrast, PR refed rats exhibited the same number of pmTOR labelled cells than their *ad libitum* fed counterparts (Figure 5F and 5G).

### Effect of perinatal protein restriction on mTOR signalling in POMC neurons

**Figure 6:**
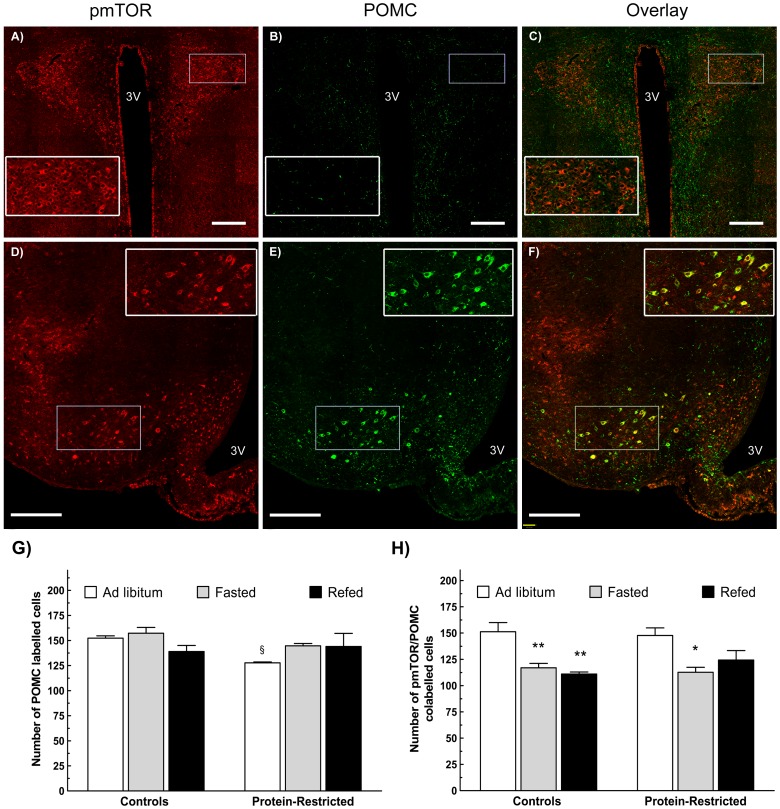
Effect of early protein restriction on mTOR phosphorylation within hypothalamic POMC-expressing neurons. Panels correspond to representative images of pmTOR and POMC labeling in the PVN (A, B and C), or the mediobasal hypothalamus (D, E and F), of *ad libitum* fed control rats. The bottom graphs correspond to the mean number of POMC cells (G) or POMC/pmTOR colabelled cells (H) in the fed state or after fasting and fasting plus re-feeding. 3 V, third ventricle. ^§^P<0.05 compared to *ad libitum* fed control animals (Student's t test). *P<0.05; **P<0.01 in relation to *ad libitum* fed animals from the same group (One way ANOVA followed by Dunnett's test). n = 3 for all groups. Scale bar  = 300 µm for all pictures.

To ascertain that the changes in the phosphorylation of mTOR in response food availability were taking place in neuronal cells involved the regulation of food intake and energy homeostasis, a subset of brain sections were subjected to double immunolabelling using a combination of pmTOR and POMC antibodies as described in the Materials and Methods section. Using this protocol, POMC and pmTOR cells could clearly be observed in the mediobasal hypothalamus where the cells bodies of the POMC neurons are located (Figure 6D, 6E and 6F). In contrast, only pmTOR-labelled cells were observed in the paraventricular nucleus (Figure 6A, 6B).

In the non fasted state, PR rats exhibited a reduced number of POMC cells (Figure 6G). Nevertheless, the number of POMC/pmTOR co-labelled cells was the same in both groups (Fig. 6H). Likewise, the number of POMC cells expressing pmTOR decreased in the same proportion in control and PR animals after fasting (Figure 6H).

## Discussion

Given its prominent role in the regulation of energy homeostasis, the hypothalamus has been the subject of several studies aimed at establishing the mechanisms of the developmental programming of obesity and related disorders. These studies have demonstrated that perinatally malnourished rats develop hyperphagia [18], [19] which is associated with impaired hypothalamic expression of orexigenic and anorexigenic genes [31], [32] and with a reduction of the inhibitory effects on feeding of insulin [33], leptin [5], [34] and serotonin [35]. Central to the detection and integration of nutrient-related information in the hypothalamus, is the protein kinase mTOR, a downstream target of a large range of neural and hormonal signals regulating food intake and energy homeostasis. Actually, hypothalamic mTOR modulates the anorexic action of ciliary neurotrophic factor (CNTF) and leptin [28], [36], and acute variations in the nutritional state of the organism induce important modifications on hypothalamic mTOR activity [28], [37]. Notably, fasting inhibits mTOR in the arcuate nucleus while in response to nutrient supply mTOR is activated [28]. Moreover, the expression of a constitutively active form of S6K1, a downstream effector of mTOR, in the mediobasal hypothalamus results in hypophagia whereas a dominant-negative form of S6K1 increases food intake [38]. Therefore, increased hypothalamic mTOR signalling is generally associated with decreased feeding.

The present results show that adult rats born to dams fed a low protein diet during gestation and lactation exhibit enhanced activation of hypothalamic mTOR in the fed state as well as impaired mTOR responses to fasting and re-feeding that differ from one hypothalamic nucleus to another. Thus, under *ad libitum* feeding conditions PR rats exhibited decreased numbers of phosphorylated rpS6 and pmTOR immunostained cells in the VMH and ARC but increased numbers of pmTOR immunopositive cells in the PVN. Moreover, the phosphorylation of rpS6 and mTOR in the VMH decreased with fasting in control but not in PR animals and, conversely, fasting decreased the phosphorylation of mTOR in the PVN of malnourished but not of control rats. These results are in line with previous observations showing differential regulation of mTOR within the hypothalamus in response to fasting or leptin administration [28], [37] but seem to be at odds with the hyperphagic phenotype of early malnourished rats documented by previous studies [18], [31]. It should be mentioned, however, that perinatal malnutrition induces only a transient increase in feeding. Actually, at the age at which the present analysis was performed, PR rats exhibit no differences in *ad libitum* food intake in relation to controls [31]. Moreover, the constitutive activation of mTOR in the hypothalamus, specifically in POMC neurons, promotes food intake and obesity [39]. To explain the discrepancies between the effects induced by the pharmacological and the constitutive activation of mTOR, it has been proposed that the ultimate effects of this kinase on food intake might depend on the cell type on which it is activated [39]. We therefore evaluated the phosphorylation of mTOR in POMC expressing neurons in the ARC by double immunofluorescence. There were no differences in the number of POMC/pmTOR co-labelled cells between control and PR rats in the fed state and both groups exhibited a statistically significant decrease in the activation of mTOR in POMC expressing neurons in response to fasting. These results indicate that early protein-restriction does not alter the nutrient sensing function of mTOR in POMC neurons but do not rule out the role of the hypothalamic POMC system as target of metabolic programming. Actually, we observed a decreased number of POMC cells in the ARC of PR animals in relation to controls. Interestingly, we and other authors have previously documented that PR rats exhibit decreased hypothalamic expression of POMC [31], [40]. The present immunofluorescence results therefore suggest that these changes might be the consequence of a reduced number of POMC cells and not the result of decreased POMC-gene expression.

The activity of mTOR is submitted to insulin regulation trough the PI3K/Akt signalling pathway. Indeed, the binding of insulin to its cell-membrane receptors promotes the activation of PI3K through the recruitment of insulin receptor substrate (IRS). The phosphorylated form of PI3K then activates Phosphoinositide-dependent kinase-1 (PDK1. The activated form of PDK1 in turn phosphorylates the serine threonine kinase Akt which stimulates mTOR by relieving the inhibitory effect of tuberous sclerosis complex 1 and 2 (TSC1/TSC2), or by promoting the phosphorylation and dissociation of proline-rich Akt/PKB substrate 40 kDa (PRAS40) from mTOR [41], [42]. In agreement with previous studies [43], [44], we observed that in the fed state PR rats exhibited decreased concentration of insulin in plasma along with normal levels of glucose and enhanced AKT phosphorylation. Normal blood levels of glucose in the presence of low insulin are indicative of increased insulin sensitivity [45], [46] and, as a matter of fact, previous studies have demonstrated that young adult rats born to protein-restricted mothers show improved glucose uptake into muscle and adipose tissue after an insulin load [47], [48], increased expression of insulin receptors both in skeletal muscle and adipocytes [47], [48], and enhanced expression and phosphorylation of AKT [43]. The increased activation of mTOR in the paraventricular nucleus of *ad libitum* fed PR rats could therefore be explained by altered PI3K-Akt signalling resulting from an enhanced tonic stimulation of insulin receptors. However, this interpretation does not fit the data obtained in the arcuate nucleus which show, on the contrary, decreased activation of mTOR as indicated by the reduced number of phosphorylated rpS6 and pmTOR-immunostained cells. Rather, we believe that the divergent activation levels of mTOR in the ARC and PVN might indicate that refeeding alters mTOR independently of the insulin-signalling pathway and/or that insulin is not the only factor involved in the activation of mTOR in response to fasting.

In summary, our data indicate that mTOR can be altered in the long term by a transient nutritional insult during early life. The alterations of mTOR occur at a developmental stage at which perintallly-undernourished animals do not show yet obesity or glucose intolerance. These observations suggest that altered nutrient sensing in response to an inadequate foetal and neonatal energetic environment is one of the basic mechanisms of the developmental programming of metabolic disorders and might play a causing role in the development of the metabolic syndrome induced by malnutrition during early life. Given the key position of mTOR at the crossroads of several hormonal and nutrient signalling pathways regulating food intake and energy homeostasis, this kinase might constitute a major gatekeeper system underlying the developmental programming of metabolic disorders.

## Supporting Information

Figure S1 Glucose tolerance test of adult rats born to control dams or born to dams fed a low protein diet during gestation and lactation. The inset shows the glucose area under the curve (AUC) during glucose tolerance test.
(TIF)

Figure S2 Changes in mTOR activity within the arcuate nucleus of the hypothalamus in response to variations in nutrient supply in adult rats born to control or protein-restricted dams. Top panels correspond to representative images of pmTOR labeling in hypothalamic slices from control (A,B,C) or perinatally undernourished rats (D,E,F) sacrificed under *ad libitum* feeding conditions, after a fast of 48 h or after fasting followed by re-feeding. Bottom graph (G), corresponds to the mean number of immune-positive pmTOR cells in the fed state or after fasting and fasting plus re-feeding. 3 V, third ventricle. ^§^p<0.05 as compared to *ad libitum* fed control animals (Student's t-test). *P<0.05 in relation to *ad libitum* fed animals from the same experimental group (One way ANOVA followed by Dunnett's test). n = 5 for all the groups. Scale bar  = 300 µm for all pictures.
(TIF)
