# Supplementary figures and images for: Impaired Hypothalamic mTOR Activation in the Adult Rat Offspring Born to Mothers Fed a Low-Protein Diet
Source: PLoS One. 2013 Sep 9;8(9):e74990. doi: 10.1371/journal.pone.0074990 (PMC3767644; doi:10.1371/journal.pone.0074990)

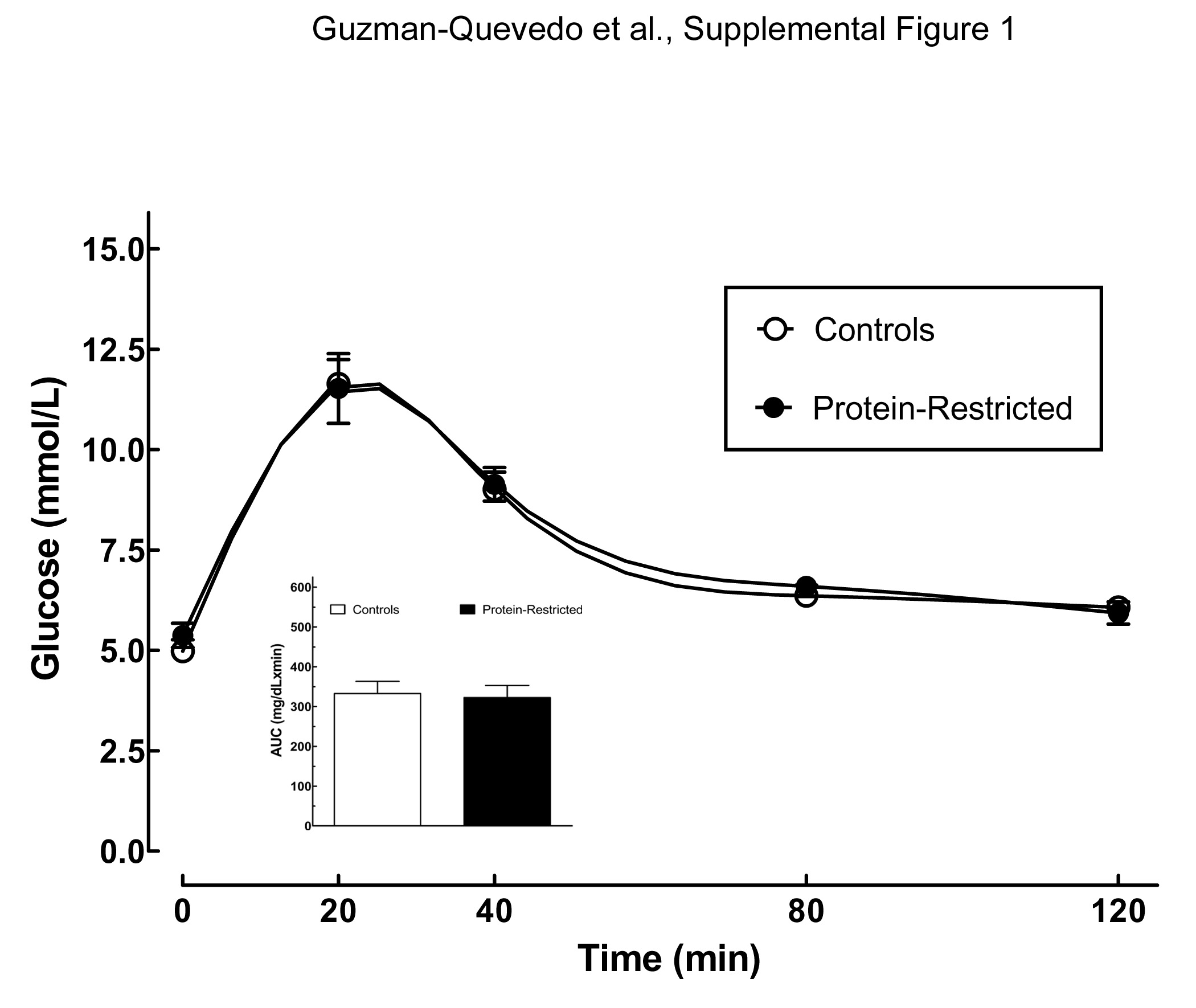

Supplement: Figure S1 — Glucose tolerance test of adult rats born to control dams or born to dams fed a low protein diet during gestation and lactation. The inset shows the glucose area under the curve (AUC) during glucose tolerance test. (TIF) [file pone.0074990.s001.tif]

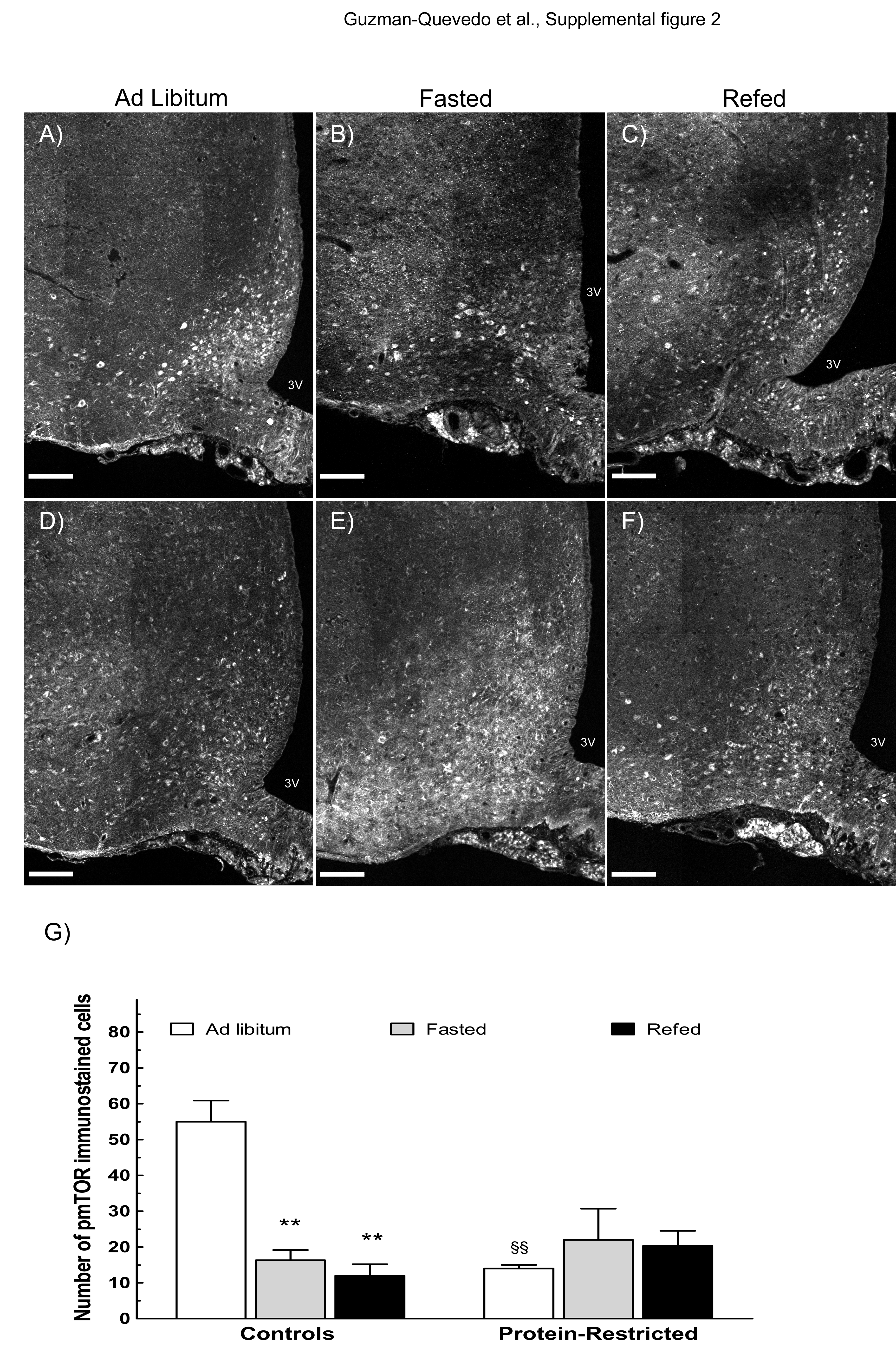

Supplement: Figure S2 — Changes in mTOR activity within the arcuate nucleus of the hypothalamus in response to variations in nutrient supply in adult rats born to control or protein-restricted dams. Top panels correspond to representative images of pmTOR labeling in hypothalamic slices from control (A,B,C) or perinatally undernourished rats (D,E,F) sacrificed under ad libitum feeding conditions, after a fast of 48 h or after fasting followed by re-feeding. Bottom graph (G), corresponds to the mean number of immune-positive pmTOR cells in the fed state or after fasting and fasting plus re-feeding. 3 V, third ventricle. §p<0.05 as compared to ad libitum fed control animals (Student's t-test). *P<0.05 in relation to ad libitum fed animals from the same experimental group (One way ANOVA followed by Dunnett's test). n = 5 for all the groups. Scale bar = 300 µm for all pictures. (TIF) [file pone.0074990.s002.tif]
